# Supplementary material for: Colloidal quantum dot molecules manifesting quantum coupling at room temperature
Source: Nat Commun. 2019 Dec 16;10:5401. doi: 10.1038/s41467-019-13349-1 (PMC6915722; doi:10.1038/s41467-019-13349-1)
Supplement: Supplementary file 1 — Supplementary Information [file 41467_2019_13349_MOESM1_ESM.pdf]

Supplementary Information for

Colloidal Quantum Dot Molecules Manifesting Quantum Coupling at Room Temperature

Jiabin Cui[†], Yossef E. Panfil[†], Somnath Koley[†], Doaa Shamalia, Nir Waiskopf, Sergei Remennik, Inna Popov, Meirav Oded & Uri Banin*

† These authors contributed equally to this work.

* Corresponding author. Email: uri.banin@mail.huji.ac.il (U.B.).

Contents:

Supplementary Discussion

Supplementary Figure 1 to 24

Supplementary Tables 1 to 3

Supplementary References

Supplementary Discussion

Electronic structure calculation methodology: We have calculated the energy levels and the electron and hole wave-functions of the CdSe/CdS monomer and dimers using the multiphysics mode of COMSOL.[1,2] Electron and hole states are calculated with a 3D single-band effective mass Hamiltonian. Interacting electron and hole states are obtained by iterative solution of the Schrödinger-Poisson equations, within a self-consistent Hartree procedure, taking into account the dielectric mismatch between the surroundings and the CQD. The dimensions we used in the calculation are based on experimental data. Material-dependent parameters such as effective masses $m^*_{e,h}(r)$ dielectric constants $\varepsilon(r)$, and conduction and valence band profiles ($V^{e,h}_{confinment}$) used in this calculation are summarized in Supplementary Table 1. The entire computational space extends further from the QD boundary allowing for electron and hole wavefunctions to extend outside of the QD boundaries and decay into free space.

We start the simulation by computing the non-interacting electron and hole states by solving the Schrödinger equation:

$$\left(-\frac{\hbar^2}{2}\nabla\left(\frac{1}{m^*_{e,h}(r)}\nabla\right)+V^{e,h}_{confinment}(r)\right)\Psi^{n}_{e,h}=E^{n}_{e,h}\Psi^{n}_{e,h}(r) \qquad (1)$$

We use von Neumann boundary-condition at the inner (between core-shell) and outer boundaries of the QD in order to impose the Ben-Daniel-Duke condition. At the edge of the computational domain (around 100 nm away from the QD) we set the Dirichlet boundary-condition by setting the wavefunction to zero. After normalization of the wavefunctions, the Poisson equation is solved to derive the hole or electron Coulombic potential generated from the other particle $\phi_{e,h}(r)$.

$$\nabla(\varepsilon_0\cdot\varepsilon(r)\nabla\phi_{e,h}(r))=q_e<\Psi_{e,h}(r)|\Psi_{e,h}(r)> \qquad (2)$$

With these potentials, Schrödinger equations are solved again for the electron and the hole with all of the contributions to the potentials:

$$\left(-\frac{\hbar^2}{2}\nabla\left(\frac{1}{m^*_{e}(r)}\nabla\right)+V^{e}_{confinment}(r)+q_e\cdot\phi_h(r)\right)\Psi^{n}_{e}(r)=E^{n}_{e}\Psi^{n}_{e}(r) \qquad (3)$$

$$\left(-\frac{\hbar^2}{2}\nabla\left(\frac{1}{m^*_{h}(r)}\nabla\right)+V^{h}_{confinment}(r)+q_e\cdot\phi_e(r)\right)\Psi^{n}_{h}(r)=E^{n}_{h}\Psi^{n}_{h}(r) \qquad (4)$$

This process is repeated iteratively until the electron and hole energies converge. In most cases, three iterations are sufficient to obtain a convergence. The emission energy was calculated by:

$$E_{emission} = E_g + E_e^i + E_h^i - E_{coulomb} \quad (5)$$

$$E_{coulomb} = \frac{E_e^i - E_e^f + E_h^i - E_h^f}{2} \quad (6)$$

Where E_g (1.76 eV) [3] is the energy gap of CdSe. $E_{emission}$ is calculated in a way which avoids the consideration of the coulomb potential twice (both for the electron and both for the hole).

Supplementary Table 1. Material parameters used in the simulations.

	CdSe	CdS	Environment	Units	Ref.
$V^e_{confinment}$	0	0.1	5.1	[eV]	(3)
$V^h_{confinment}$	0	-0.64	-5.64	[eV]	(3)
m_e^*	0.112	0.21	1	m_0	(4)
$m_{h\perp}^*$	0.48	0.376	1	m_0	(4)
$m_{h\parallel}^*$	1.19	0.746	1	m_0	(4)
$\varepsilon_\perp$	9.29	8.28	1	-	(4)
$\varepsilon_\parallel$	10.16	8.73	1	-	(4)

Consideration of Biexciton quantum yield (QY) to exciton QY ratio in fused dimers: To consider the obtained *g2*(0) value as the biexciton quantum yield, we kept the excitation power significantly low so that the average number of excitons produced per pulse (<N>) always remained below 0.2 [5].

The average number of excitons produced per pulse is given by the formula $< \mathrm{N} >= \frac{\sigma J}{E_{Photon} \cdot A}$ where σ is the absorption cross section of the particle at the excitation wavelength (375 nm in our case), J and E_{Photon} are the pulse energy and photon energy of the excitation light, respectively, and A is the spot area. The absorption cross-section was calculated according to the procedure reported elsewhere. [6] The value of the absorption cross-section for the 1.4/2.1 nm CQDs and the corresponding fused dimer at 375nm was found to be 2.3×10^{-15} cm^2 and 5.7×10^{-15} cm^2, respectively.

Excitation power dependence study on single fused dimer: The flickering nature of the fluorescence in single fused dimers associated with a tri-exponential lifetime decay (comprising of ~2 ns, ~5 ns and ~25 ns components) was observed for the 1.4/2.1 Coupled CQDs. The fluorescence flickering nature was present upon changing the laser power and observed even at the lowest measurable excitation. The intensity traces followed a single distribution of intensities (much narrower than the high excitation) without reaching an off state (Supplementary Figure 24). Upon excitation of the particle by a short pulse laser, a charge state of the exciton can be created along with the neutral exciton which can contribute to the appearance of short decay components [7, 8].

For the 1.4/2.1 nm monomer CQDs the contribution of the short fluorescence decay component was found to be negligible at similar excitation condition, whereas the decay for dimers comprised of these CQDs was dominated by the short components. With decrease in the excitation intensity, we observed an enhancement in the contribution of the ~25 ns component on account of the ~2 ns and ~5 ns components (Fig. 5f in the main manuscript). This clearly indicates on the enhanced photo-charging effect in case of fused dimers. Associated with the flickering of the intensity, we observed a lifetime distribution through the intensity traces (lower intensity traces have shorter lifetime as shown in figure 5c). Upon the decrease in photo-charging not only the average lifetime increases (from 4.5 ns at <N>~0.18 to 7.2 ns at <N>~0.03), but also the lifetime distribution (commonly referred as lifetime flickering) narrows down.

Formation of coupled CQDs molecule dimers

We utilized silica nanoparticles as a template [9] for the fabrication of coupled CQD molecules. The detailed route-line is depicted in Supplementary Figure 1. This was performed by the following steps:

1. Fabrication of SiO_2 nanoparticles, coated by MPTMS. This kind of SiO_2 particle presents thiol groups on its outer surface, which are later used for the binding of the CQDs.
2. Core/shell CQDs binding to the SiO_2 particle surface: mixing a solution of the chosen core/shell CQDs with the SiO_2 nanoparticles allows their binding to the available thiol sites on the silica surface.
3. Growth of a secondary thin layer of SiO_2 on the QDs@ SiO_2 for masking: In this manner the CQDs are immobilized and cannot rotate or reorient while only a top hemisphere is remained exposed for further chemical functionalization of the CQDs.
4. Selective surface decoration of the CQDs by linker groups: Chemical grafting of a functional structure based on thiol group as linkers is then applied which selectively reacts only with the exposed NC hemisphere. Here, a tetrathiol ligand was added as a linker by a ligand exchange reaction on the exposed CQDs surface (for example oleyamine).
5. Forming the dimer geometry on the silica surface: Addition of a solution with the second CQDs allows their conjugation, yielding a controlled formation of a dimer structure through the binding linker.
6. Dimer release: The dimers are released from the silica surface and separated. Here, this can be achieved by selective SiO_2 etching using an HF/NMF solution.
7. Fusion to form the coupled CQD molecule: The dimers are fused by addition of a suitable precursor and heated to form a continuous link between the two shell regions of the pre-made dimers.

Additional optional purification (size selective separation) of the dimers from free monomer and higher linked oligomers is possible in between steps 6 and 7, or after the fusion step.

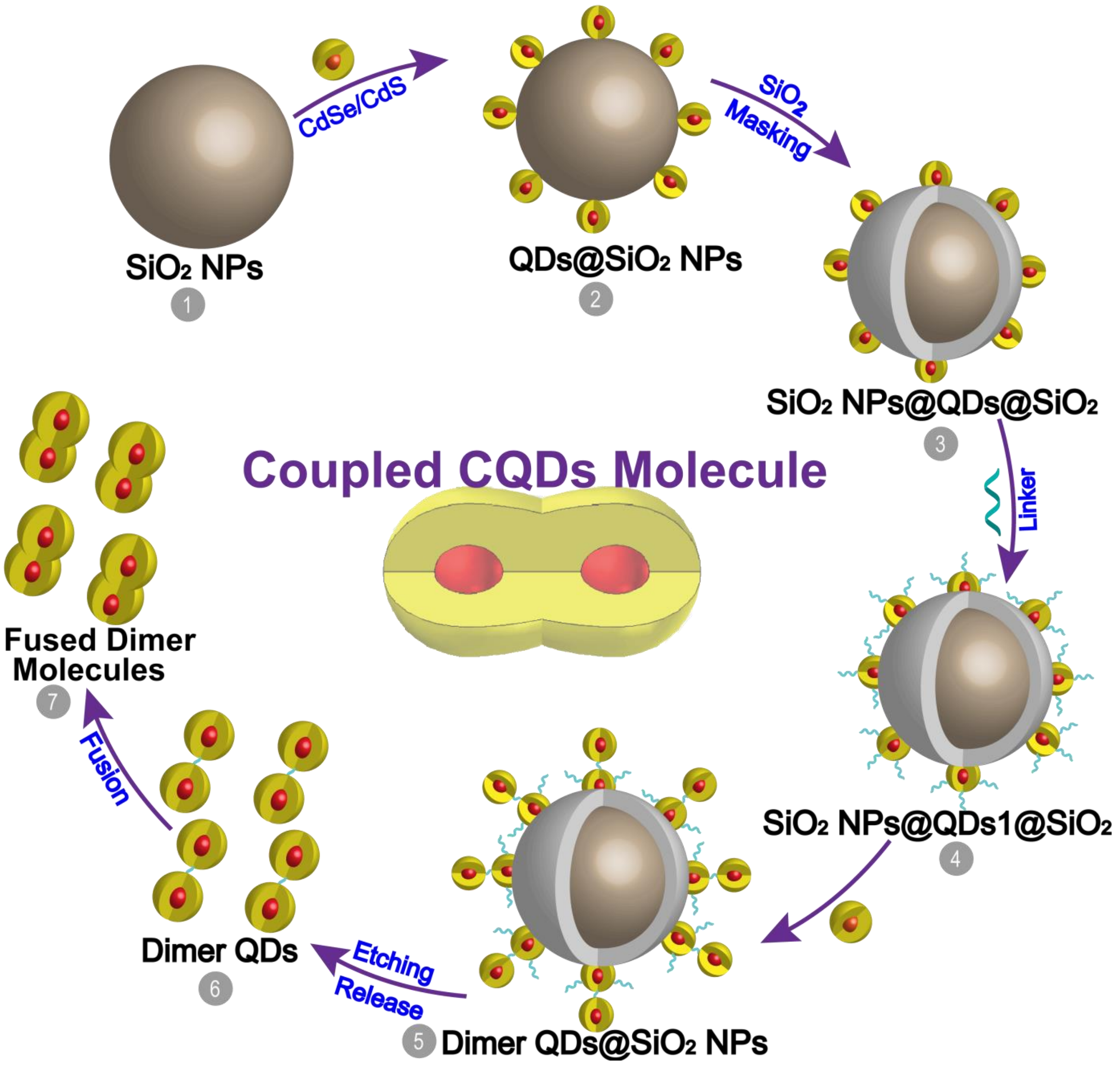

Supplementary Figure 1. Scheme of the coupled CQDs molecule fabrication steps. Step 1: SiO_2 nanoparticles synthesis. Step 2: core/shell CQDs binding to the SiO_2 particle surface. Step 3: masking by a secondary thin layer of SiO_2 growth on the QDs@SiO_2. Step 4: selective surface decoration of the CQDs by linker groups. Step 5: dimer geometry formation on the silica surface. Step 6: dimer release. Step 7: fusion to form coupled CQD molecules.

CdSe@CdS Core/Shell NCs synthesis and characterization:

Supplementary Figure 2 shows electron microscopy images and the absorption and emission spectra for the different nanocrystal building blocks which were used in this work. For example, Supplementary Figure 2a-c, show CdSe cores (1.9 nm, radius) which were synthesized by the hot-injection method. The absorption (first exciton peak at 581 nm) and photoluminescence (PL) spectra (peak at 597 nm) were measured after purification. The CdSe@CdS CQDs (1.9/4.0 nm) were fabricated by CdS shell growth using the injection of Cd-oleate and octanethiol precursors. The PL spectrum (637 nm) after shell growth, shows a red-shift compared with the cores emission. This, along with the enhancement of the fluorescence quantum yield indicates the successful fabrication of quasi-type II QDs.

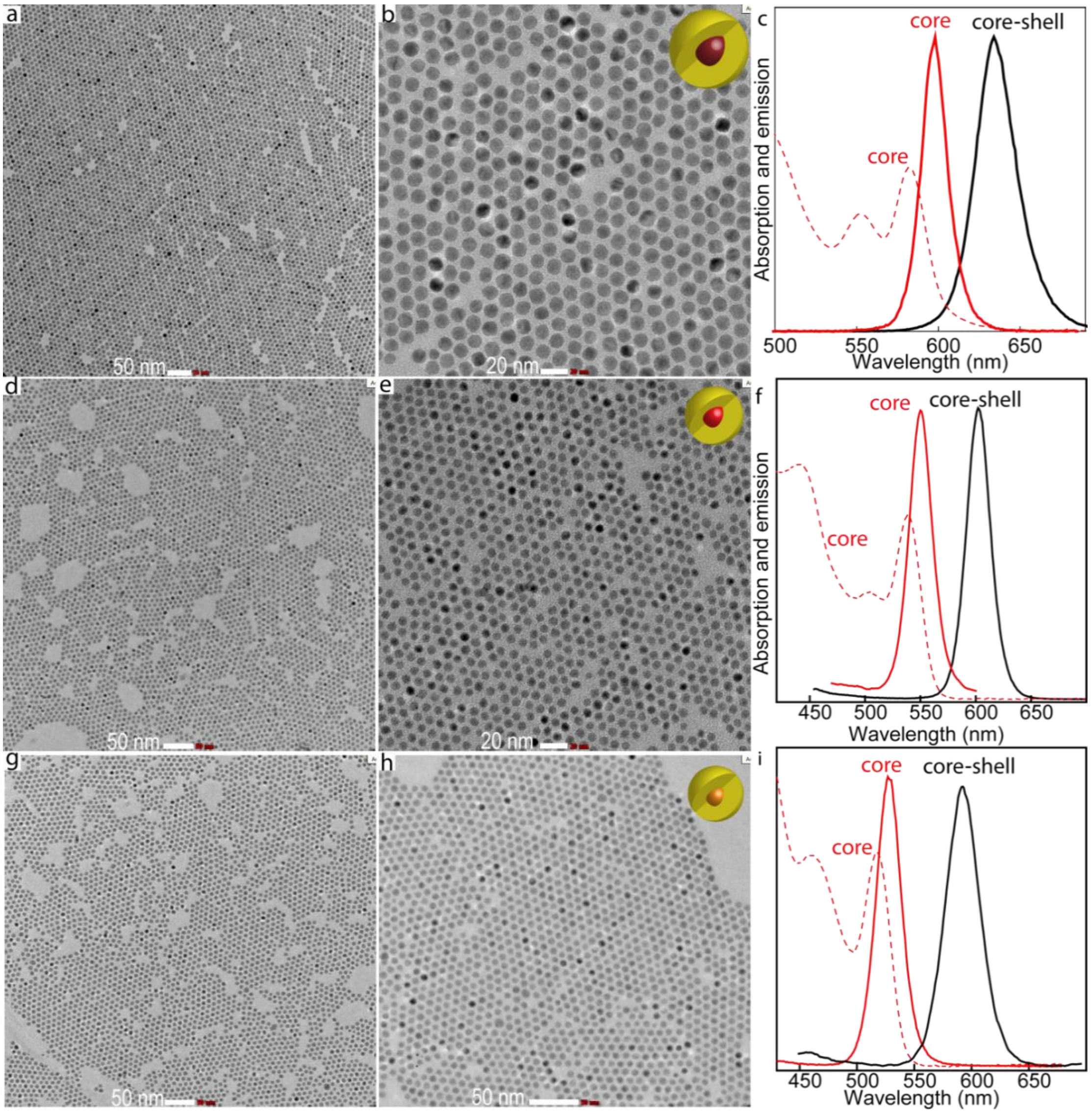

Supplementary Figure 2. Quantum dots characterization. TEM micrographs, absorption and photoluminescence spectra of different sizes of CdSe/CdS core-shell CQDs: (a-c) 1.9/4.0 nm, (d-f) 1.4/2.1 nm, and (g-i) 1.2/2.1 nm.

Structural characterization

The core/shell structure of the monomer is directly identified by atomic resolution STEM-HAADF imaging followed by fast Fourier transform (FFT) analysis (Supplementary Figure 3). Supplementary Figure 3a, b shows raw and Fourier filtered images of CQD monomers, respectively, viewed under $[\bar{1}2\bar{1}0]$ zone axes (ZA) of their wurtzite structure. At this orientation major low index atomic planes (0002), $(10\bar{1}0)$, $(10\bar{1}1)$ are clearly observed thus providing identification of boundary faces of a monomer crystal (labeled on inset in (a)). Atomic structure of CQDs at [0001] ZA with clearly visible perfect hexagonal motifs is depicted in Supplementary Figure 3g-h. The hexagonal close-packed (*hcp*) wurtzite structure of CQDs was further evidenced by the selected area electron diffraction (SAED) and X-ray powder diffraction (XRD) measurement (Supplementary Figure 3k-l).

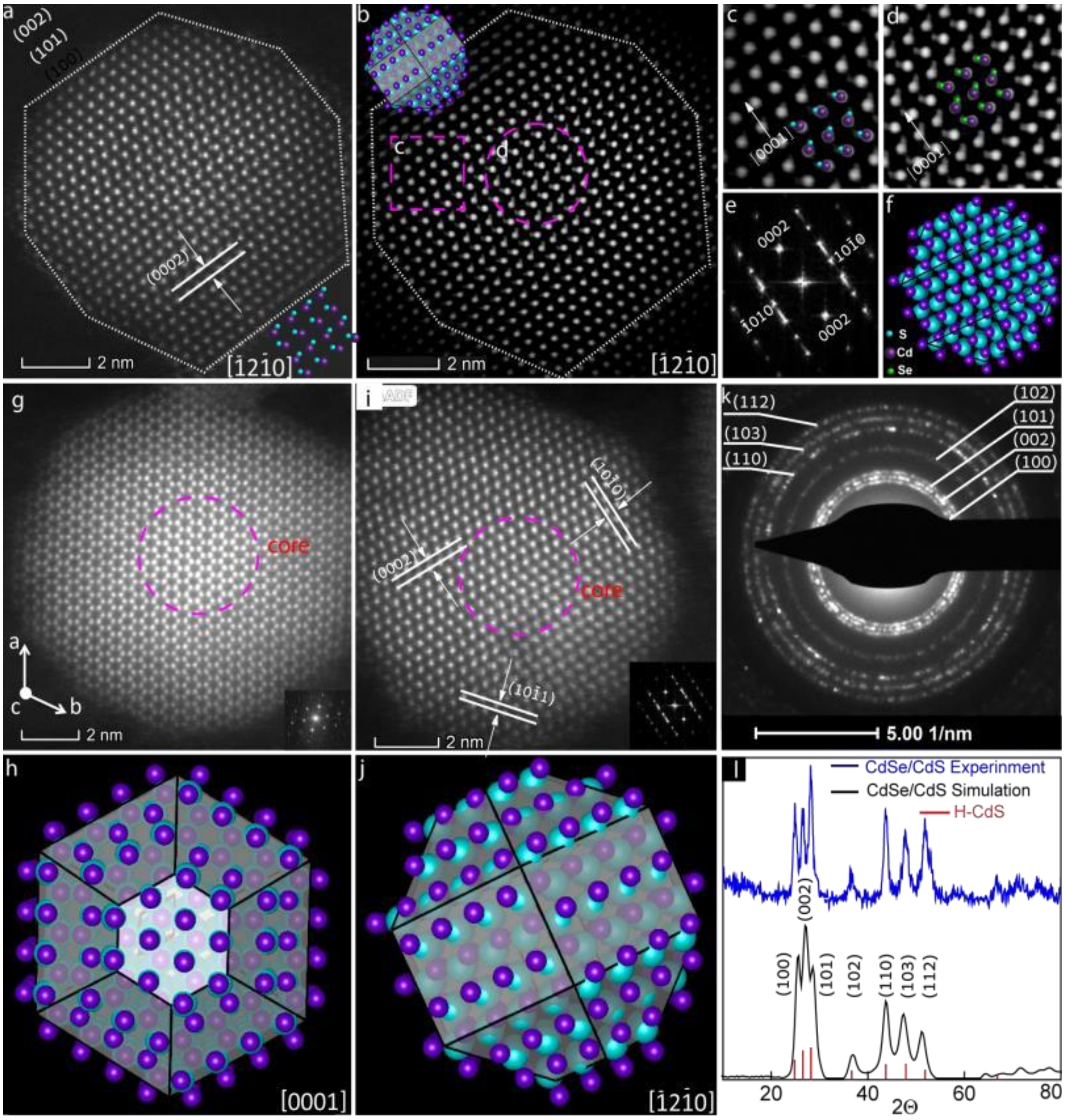

Supplementary Figure 3. Structural characterization of the coupled CQDs. Raw (a) and Fourier filtered (b) HAADF-STEM images of 1.9/4.0 nm CdSe/CdS CQD monomer viewed under $[\bar{1}2\bar{1}0]$ zone axis (ZA). Inset in (a) is a cartoon model built with VESTA software [10] with bounding faces indexed based on the STEM data. Magnified images of edge (shell) (c) and central (core) parts (d) of the CQD shown in (b). Sulfur, Selenium and Cadmium atoms are marked in blue, green and purple, respectively. Coherent growth of the shell lattice is identified. (e) and (f) are FFT and atomic structure model of (a), respectively. HAADF-STEM image of CdSe/CdS CQD under ZA [0001] (g) and atomic structure reconstruction imaging calculated for the same orientation (h). (i) High resolution HAADF-STEM image and atomic structure model (j) of CdSe/CdS CQD viewed under ZA $[\bar{1}2\bar{1}0]$. The core regions are marked with pink circles in (g) and (i). FFT patterns are inserted in (g), and (i). SAED (k) and XRD pattern acquired at large ensembles of CdSe/CdS CQDs (blue curve – experimental XRD data, red bars - theoretical positions for diffraction peaks of *hcp*) CdS (JCPDF 04-001-6853), black curve - integrated intensity of SAED (k)).

Step I - Silica nanoparticles characterization

The SiO_2 nanoparticles (step 1) were prepared as described in the methods section[11] and characterized by TEM (Supplementary Figure 4). These nanoparticles are inherently covered by thiol linkers from the MPTMS precursor allowing QDs binding at the next step. During the washing step, small amount of diluted base was utilized to avoid cascade and aggregation and to ensure a uniform binding on each SiO_2 sphere surface in the next step.

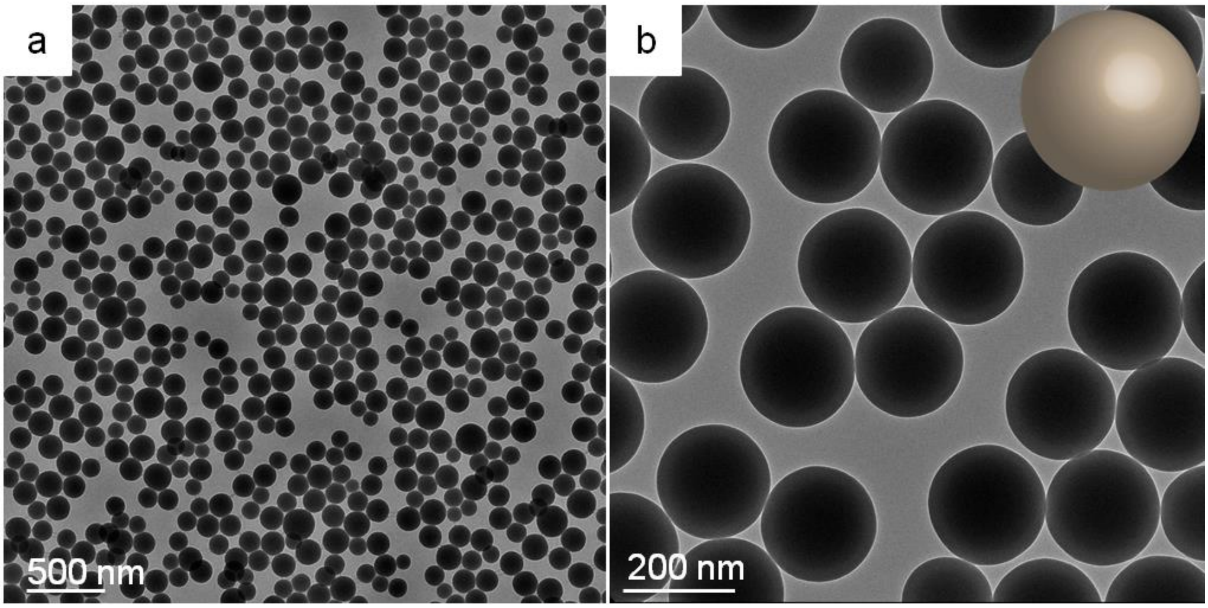

Supplementary Figure 4. TEM images of SiO_2 nanoparticles prepared by MPTMS precursor acquired at different magnifications.

Step II - Characterization of the Silica-QDs conjugates

The SiO_2@QDs particles were prepared in step 2 by adding the CdSe/CdS CQDs to the SiO_2 nanoparticles solution. The resulting particles were characterized by TEM and SEM as shown in Supplementary Figure 5. In order to avoid the CQDs overlap and aggregation on the SiO_2 surface, the ratio of CQDs added to the SiO_2 nanoparticles was controlled. In the sample below, a 1:500 SiO_2:QD ratio yielded well-separated and clearly resolved surface distribution of CQDs. The CdSe/CdS@SiO_2 nanoparticles solution was cleaned twice from free and weakly bound CQDs by centrifugation, discarding the supernatant and re-dispersion in toluene.

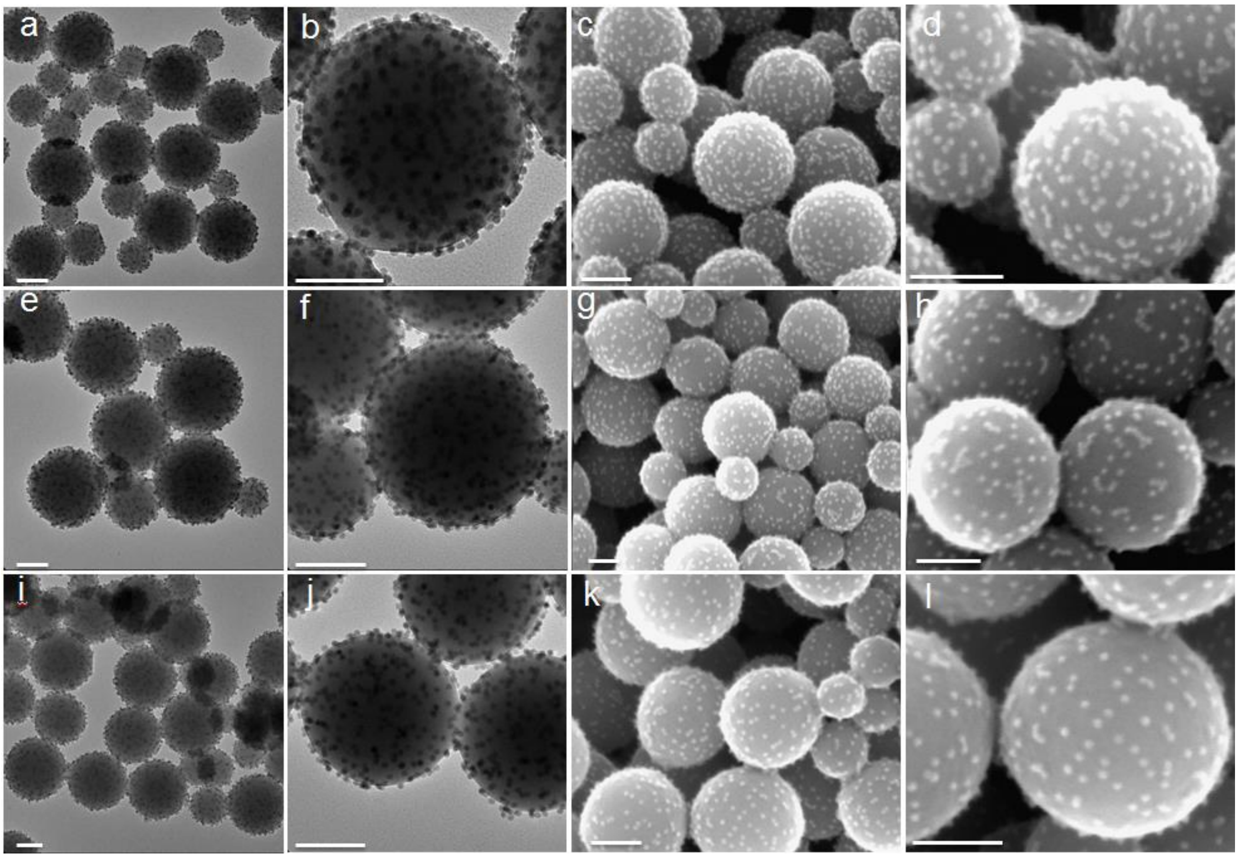

Supplementary Figure 5. CdSe/CdS@SiO_2 characterized with electron microscopy. TEM and SEM images acquired at different magnification of the CdSe/CdS@SiO_2 NPs produced with different loading ratios of (a-d) 1:2000, (e-h) 1:1000, and (i-l) 1:500. Scale bars are 100 nm.

Step III – formation of a silica masking layer

The secondary masking silica layer provides two functions: firstly, coverage of the inherent surface thiol groups of MPTMS in order to avoid the adsorption of additional CdSe-CdS CQDs in the dimerization of step 5.[11] This enhances the efficiency of the dimer formation versus monomers binding. Secondly, this immobilizes the CdSe-CdS CQDs such that they cannot rotate, exposing a hemisphere which emerges in the solvent and can be modified selectively by the chemical grafting of a functional structure/group.

In order to control the thickness of the secondary SiO_2 masking layer, an optimized mixture of PVP and TEOS was necessary. If the PVP amount is too low, it usually results in inefficient masking. Conversely, if it is too high it could lead to a full QDs masking by the SiO_2 layer, which prevents the dimer formation in the next step. Additionally, the optimization of the TEOS amount was performed as described below. According to the calculation, 50 μL of TEOS leads to a hemisphere mask. Three different amounts of TEOS were tested in our system, 200 μL (Supplementary Figure 6a-d), 100 μL (Supplementary Figure 6e-h), and 50 μL (Supplementary Figure 6i-l), respectively. For the highest TEOS loading, the CQDs were fully embedded inside the SiO_2 layer, as is clearly demonstrated in Supplementary Figure 6a-d, which is obviously useless for the next binding procedure. Instead, 50 μL of TEOS were utilized for the secondary essential minimal-limitation masking as well as providing the immobilization to ensure the dimer formation yield (Supplementary Figure 6i-l). After the growth of the secondary SiO_2 layer, the surface roughness of the CdSe-CdS@SiO_2 nanoparticles increases significantly, while the emergent CQDs can still be discerned. The TEM and SEM characterization of the resulting nanoparticles are presented in Supplementary Figure 6.

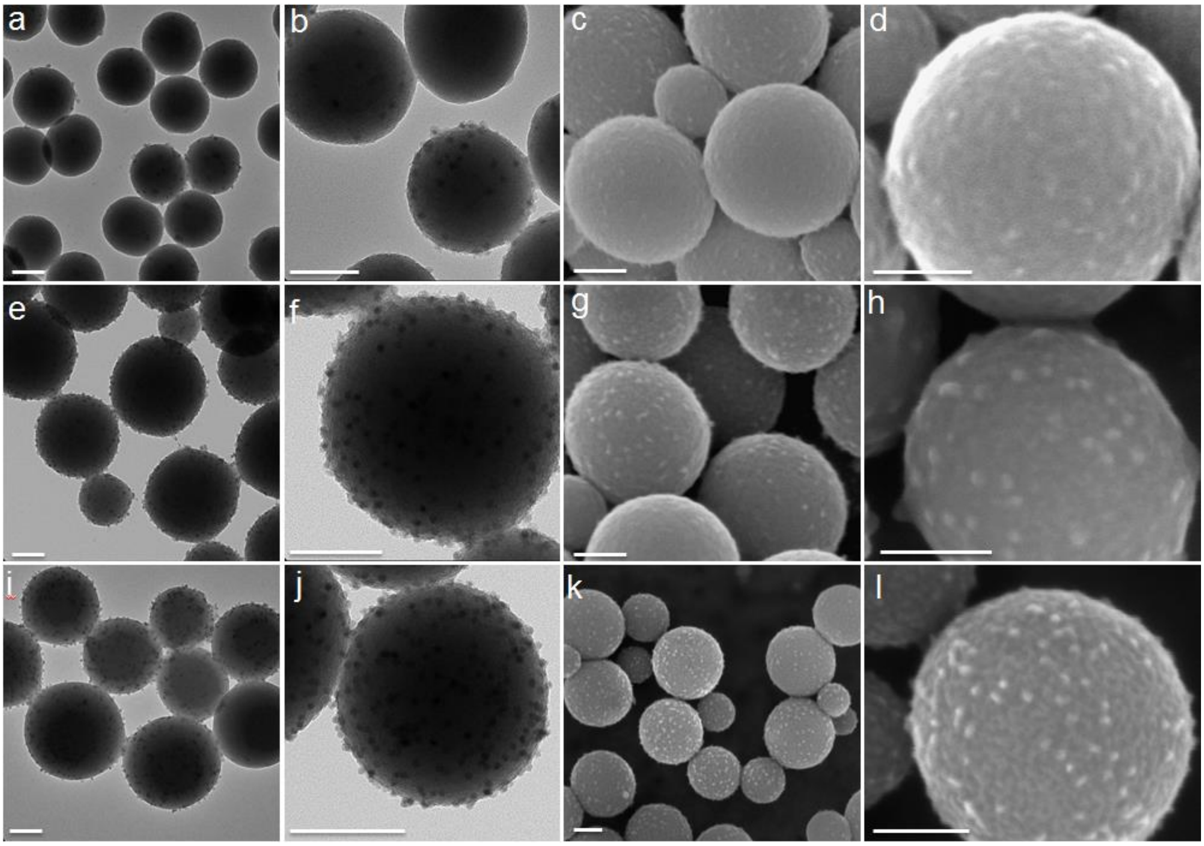

Supplementary Figure 6. TEM and SEM images acquired at different magnification of the SiO_2@CdSe/CdS@SiO_2 NPs produced with different amount of TEOS for masking (a-d) 200, (e-h) 100, and (i-l) 50 μL. Scale bars are 100 nm.

Step IV – linker binding

For the dimer formation step, the chosen linkers bind to the exposed region of the anchored CQDs (step 4). A Tetra-thiol molecule was used as a bi-dentate linker molecule (Supplementary Figure 7). The thiol groups strongly bind to the CQD surface and can displace the existing surface ligands of the exposed CQD hemisphere. In order to enhance the conjugation of the linkers, the surface modification procedure was performed under Argon flow, at 60 °C overnight. The excess linker molecules were removed by precipitation and centrifugation. Here, the cleaning step after the linker addition was significant for achieving high dimer formation yield.

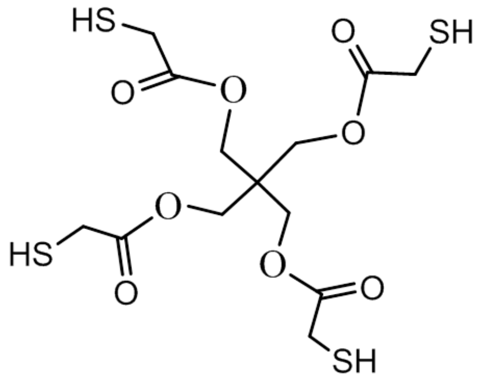

Supplementary Figure 7. Chemical structure of the linker (pentaerythritol tetrakis(3-mercapto-propionate)).

Step V – Dimer formation

For the preparation of homodimers a CQDs, a ratio of 1:1.2 to the original amount used in step 2 was employed. The resulted structures are presented in Supplementary Figure 8.

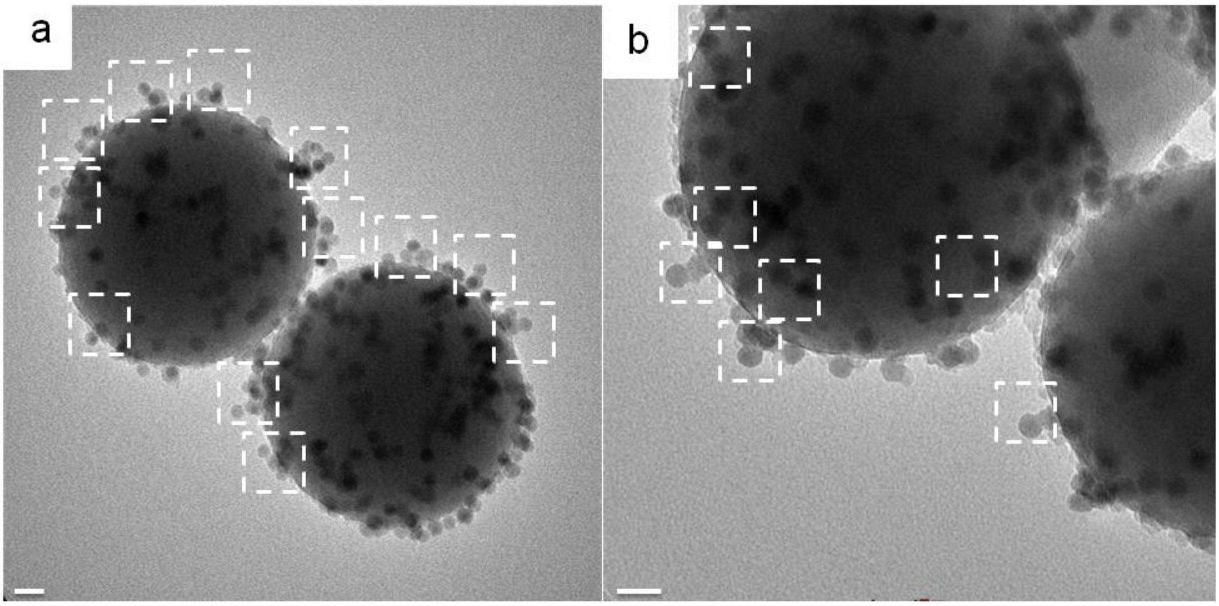

Supplementary Figure 8. TEM images of SiO_2@dimer CdSe/CdS particles acquired at different magnifications. Scale bars are 20 nm. Representative dimer structures are marked by white frames.

Step VI – Dimers release

Precisely controlled dimer CdSe-CdS CQD molecules were successfully achieved as follows. The release and separation of the CdSe-CdS CQDs dimers from the SiO_2 spheres was performed by selective etching process of the SiO_2 using an HF/NMF (10%) etching solution. The free dimers are shown in Fig. 1c. The freed dimers were separated by centrifugation decanting the supernatant and repeated ethanol precipitation/centrifugation cycles for three times.

Step VII – Fusion to form the coupled NC molecule

The fusion procedure plays a significant role in reduction of the potential barrier in the coupled CQDs molecules. The choice of correct temperature, precursor amount and ligands lead to the fusion of two CQDs without ripening and collapse.

Size selective separation process for 1.9/4.0 nm CQDs dimers

The fused dimer molecules as prepared by the procedure explained in the previous section contain some unreacted monomer CQDs which have been purified to achieve high yield of CQD molecule for further studies. Supplementary Figure 9 shows the released fused dimers, which were separated by centrifugation decanting the supernatant and repeated redispersion (ethanol)/precipitation (centrifugation) cycles for three times. Monomers were found mainly in the suspension, as shown in Supplementary Figure 9b. As seen in Supplementary Figure 9c, the monomers, along with possible higher order aggregates of CQDs can be well separated after this step, resulting in a purified solution of dimer structures.

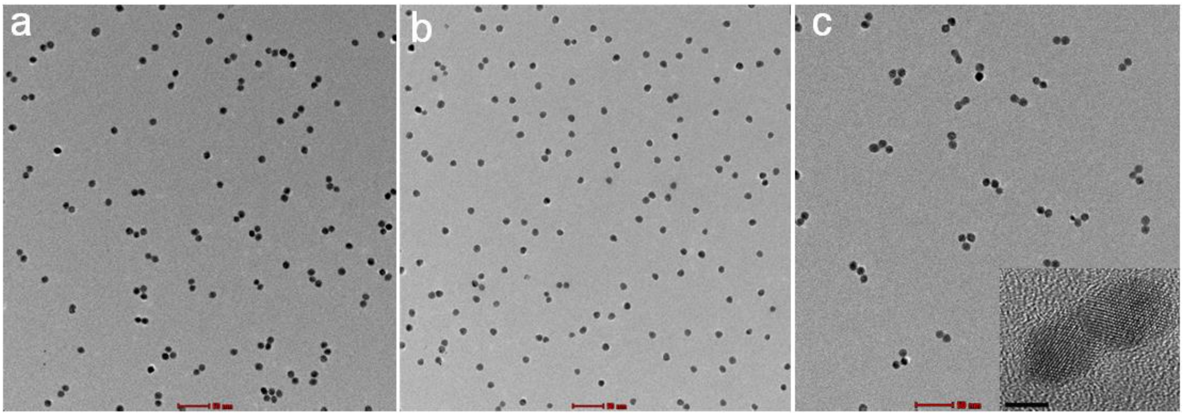

Supplementary Figure 9. TEM images of fused 1.9/4.0 nm CdSe/CdS molecules before size selective separation process (a). The suspension (b) and the precipitate (c) of fused CdSe/CdS molecules after the size selective separation process. Inset, HRTEM image of fused CdSe/CdS molecules.

Size selective separation process for 1.4/2.1 nm CQDs dimers

The size selection separation for other nanocrystal sizes is similar but may require slight modifications. For example, for 1.4/2.1 nm CdSe/CdS CQD molecules low centrifugation rate was used for efficient separation. The resulting dimers are presented in Supplementary Figure 10.

After the separation step, the sample was stored under argon atmosphere and kept in the glovebox for further characterization and single nanoparticle measurement.

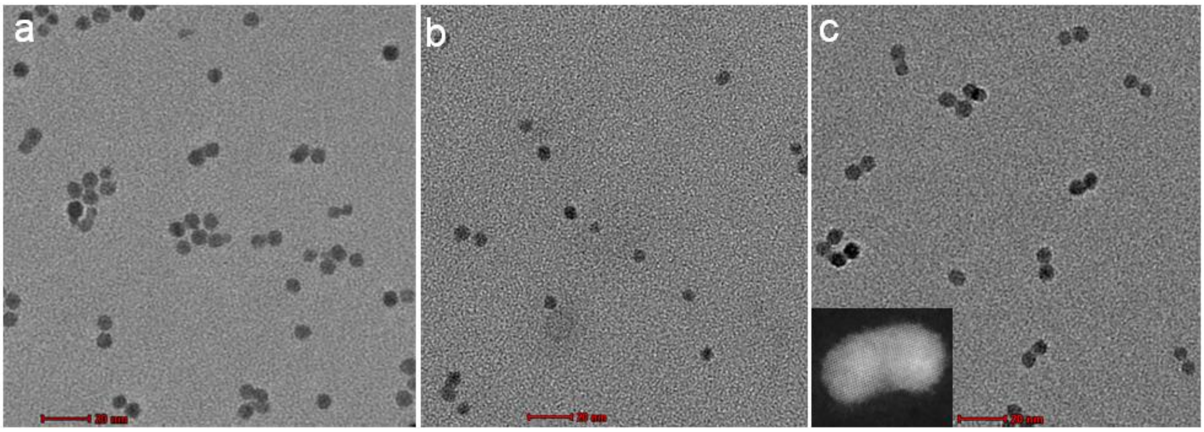

Supplementary Figure 10. TEM images of fused 1.4/2.1 nm CdSe/CdS molecules (a) before size selective separation process. The suspension (b) and the precipitate (c) of fused CdSe/CdS molecules after the size selective separation progress. Inset, HRTEM image of fused CdSe/CdS CQD molecule.

Absorption spectrum measurement for 1.4/2.1 nm CQDs

The features in the absorption spectrum change significantly after the fusion of two CQDs. To further demonstrate the changes, we have normalized the data at the bulk absorption regime (300nm). It can be observed that the band-edge absorption feature of the unfused dimer is retained and similar to the monomers. Upon fusion broadening is seen, along with a red shift and lower absorption. The excited state features are also broadened significantly upon fusion. Upon normalization at the band-edge, the significant relative change in absorption at higher energies upon fusion is emphasized (Supplementary Figure 11a). All these aspects indicate coupling effects upon fusion and formation of the CQD molecules.

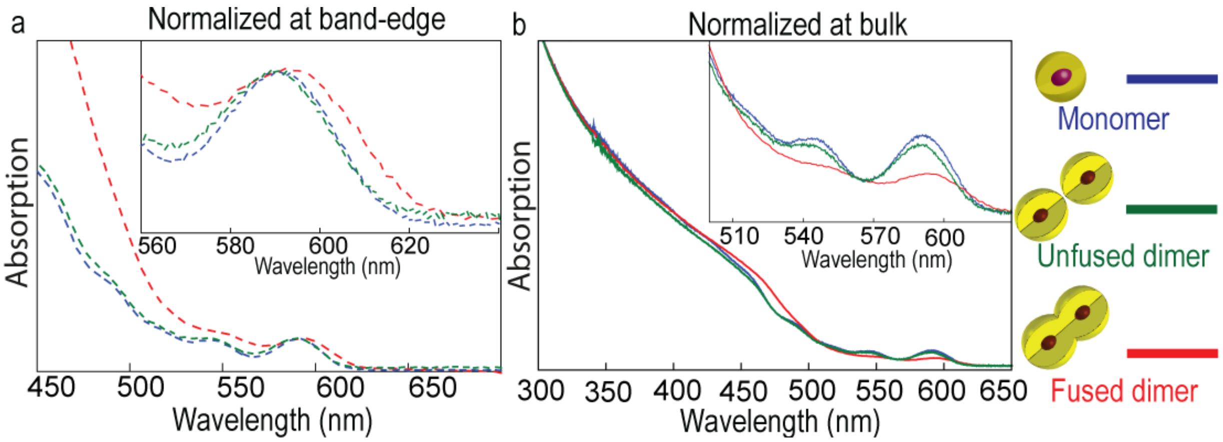

Supplementary Figure 11. The normalized absorption spectra of monomers (blue), unfused (green), and fused 1.4/2.1 nm CdSe/CdS CQD molecules when normalized with respect to (a) band-edge peak and (b) bulk transitions (300 nm).

Control experiment: monomer particles that were treated with the fusion procedure

The CQDs are exposed to different conditions during the synthetic procedure such as binding, etching, fusion etc. An important control is to identify whether the inherent properties of the CQDs change during these processes, especially during etching and fusion. Hence, we studied the absorption and photoluminescence spectra (Supplementary Figure 12) of monomers that although did not form a dimer, were still treated with the complete synthetic procedures of the fusion protocol. These monomers were separated by size selective precipitation (Supplementary Figure 10 b) and measured accordingly. The identical spectral features for the monomers, that underwent the fusion procedure, and the original ones unequivocally confirms no change in the excitonic behavior of the CQDs. This rules out the possibilities of any shift due to alloying between core/shell regions.

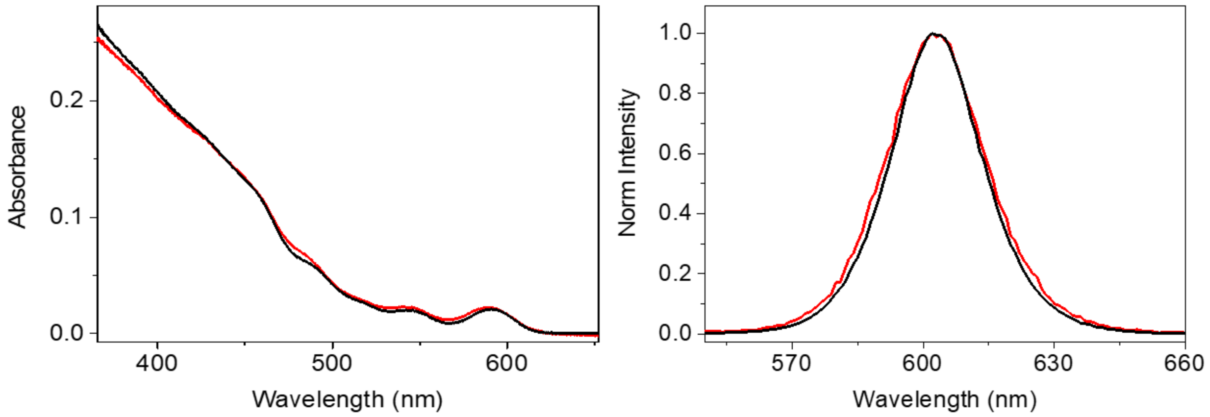

Supplementary Figure 12. The ensemble absorption and PL spectra of monomer before (black), and after the fusion procedure (red) for CdSe-CdS CQDs with core/shell size of 1.2/2.1 nm.

Spectral characterization of the various fused CQDs dimers

The red shift of the fluorescence was enhanced with a size decrease in the CdSe-CdS CQDs composing the fused dimers. That is, the smaller core-shell CQDs molecules present strong coupling properties. Additionally, upon fusion, the full width at half maximum (FWHM) measured from the fluorescence spectrum was obviously increased compared with that of the monomer sample.

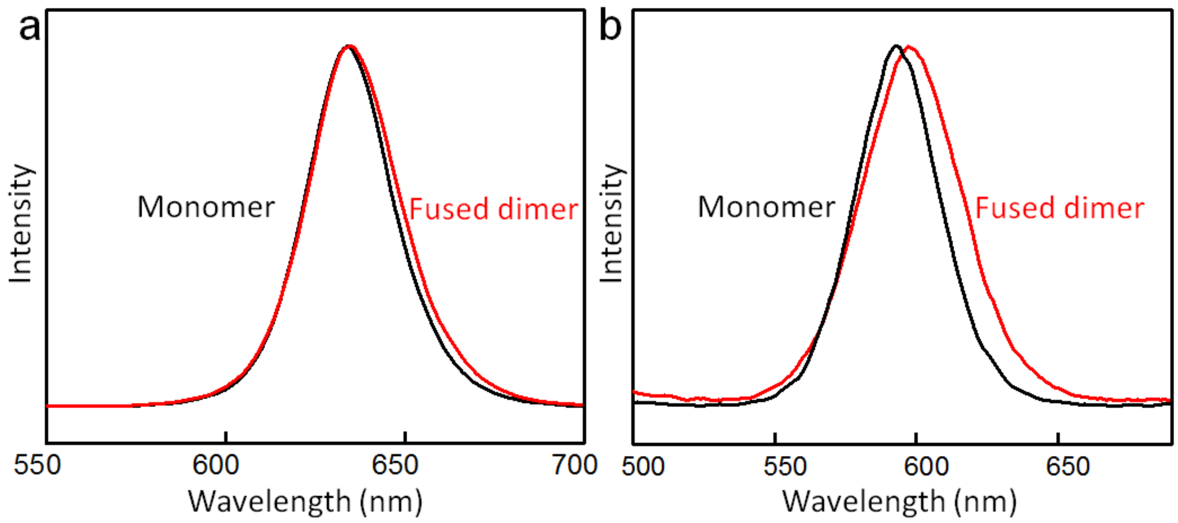

Supplementary Figure 13. The PL intensity of monomer (black), and fused (red) CdSe-CdS CQDs molecules with core/shell size of 1.9/4.0 nm (a), and 1.2/2.1 nm (b), respectively.

Structural characterization of the fused 1.9/4.0 nm CQDs dimers

Supplementary Figure 12 shows HAADF-STEM images and atomic structure models of the fused 1.9/4.0 nm CdSe/CdS CQD molecules with hetero-plane orientation relationship at attachment. The analysis allowed to determine the (0002) faces (and thus C-axis orientation) for each CQD in a pair as indicated on the images and structure models in Supplementary Figure 14. It is clearly seen that the C-axes of the monomers in a heteronymous-plane fused dimer are randomly oriented unlike the distinct parallel alignment observed at homonymous-plane attachment (see main text).

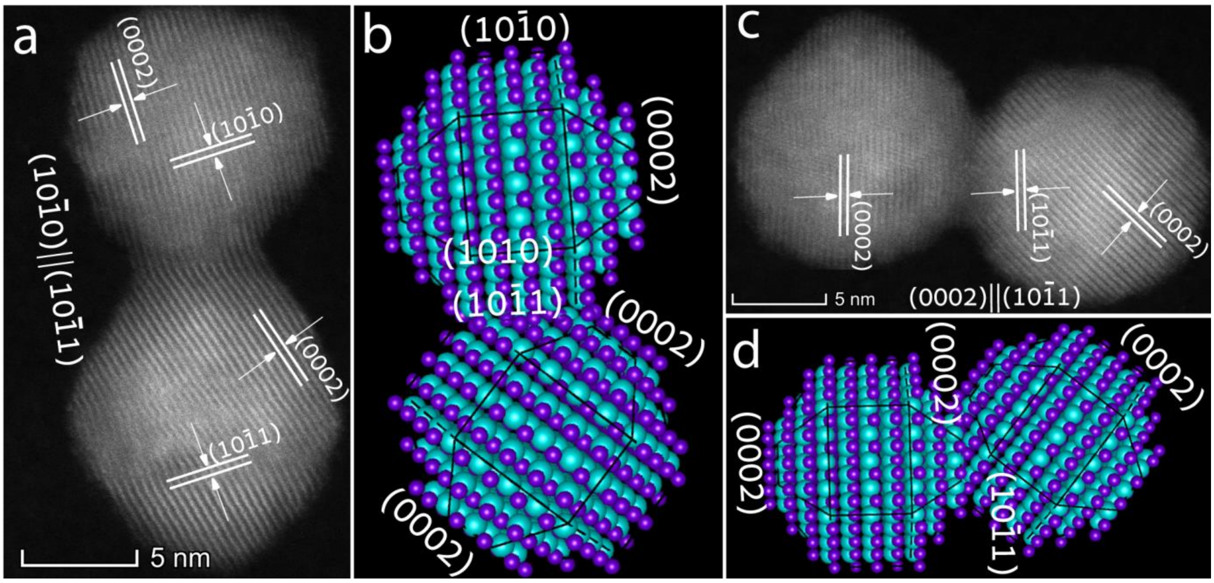

Supplementary Figure 14. HAADF-STEM images and atomic structure models of the fused 1.9/4.0 nm CdSe/CdS molecules with hetero-plane attachment of (10$\bar{1}$0)||(10$\bar{1}$1) (a-b), (0002)||(10$\bar{1}$1) (c-d). For the atomic model, the Cadmium atoms are marked in brown and Sulfur atoms in blue.

Structural characterization of the fused 1.4/2.1 nm CQDs dimers

In our system, the attachment and fusion of the monomer CdSe/CdS CQDs was based on the linker's binding. Therefore, the attachment orientation relationships for the smaller CdSe/CdS CQDs molecules were similar to those observed for the larger CQDs. Yet, a small difference was still noticed. Namely, there are three main options for homonymous-plane attachment: (0002)||(0002), $(10\bar{1}0)||(10\bar{1}0)$, and $(10\bar{1}1)||(10\bar{1}1)$. Supplementary Figure 13a shows the $(10\bar{1}0)||(10\bar{1}0)$ homonymous-plane attachment with $(10\bar{1}0)$ faces of A1 parallel to those of A2 and a continuous lattice observed through the entire dimer.

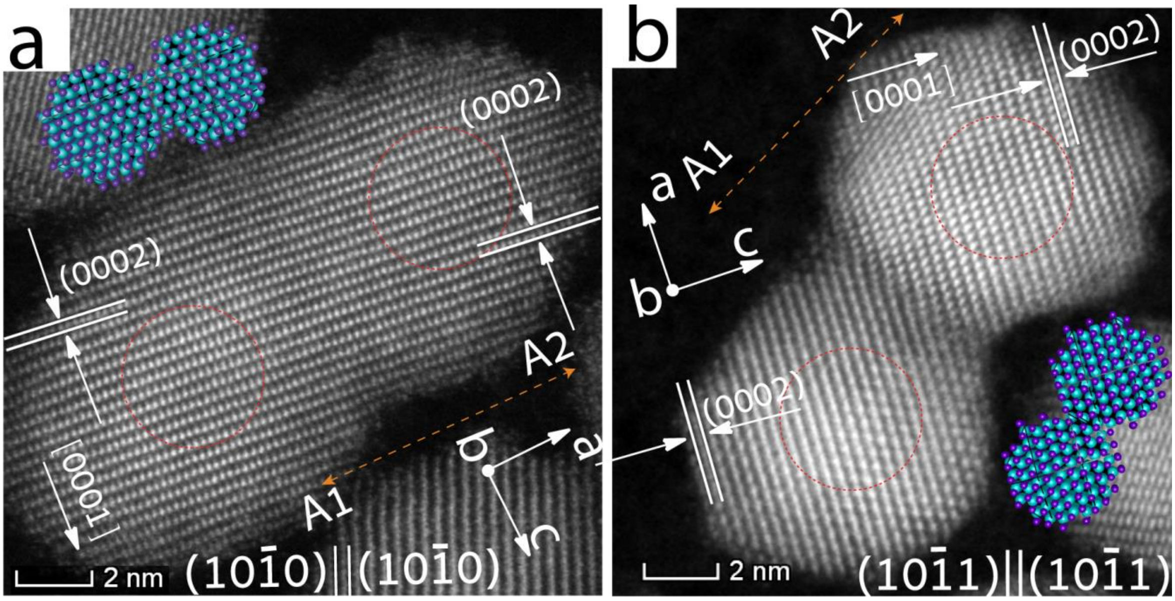

Supplementary Figure 15. HAADF-STEM image and atomic structure model of the fused 1.4/2.1 nm CdSe/CdS molecules with homo-plane attachment on $(10\bar{1}0)||(10\bar{1}0)$ (a), and $(10\bar{1}1)||(10\bar{1}1)$ (b). For the atomic model, the Cadmium atoms are marked in purple and Sulfur atoms in light blue.

Heteronymous-plane attachment of the fused 1.4/2.1 nm CQDs dimers

The hetero-plane attachment mainly based on (0002)||($10\bar{1}0$), (0002)||($10\bar{1}1$), and ($10\bar{1}1$)||($10\bar{1}0$) was also observed in the fused 1.4/2.1 nm CdSe/CdS CQD molecules. In this case, due to the thin CdS shell (only 6 atomic layers thick), the core CdSe region (Cd: atom with high brightness, Se: atom with slight brightness) was directly identified at HAADF STEM image. Supplementary Figure 14 shows examples of heteronymous-plane attachments at (0002)||($10\bar{1}0$) (a) and (0002)||($10\bar{1}1$) (b).

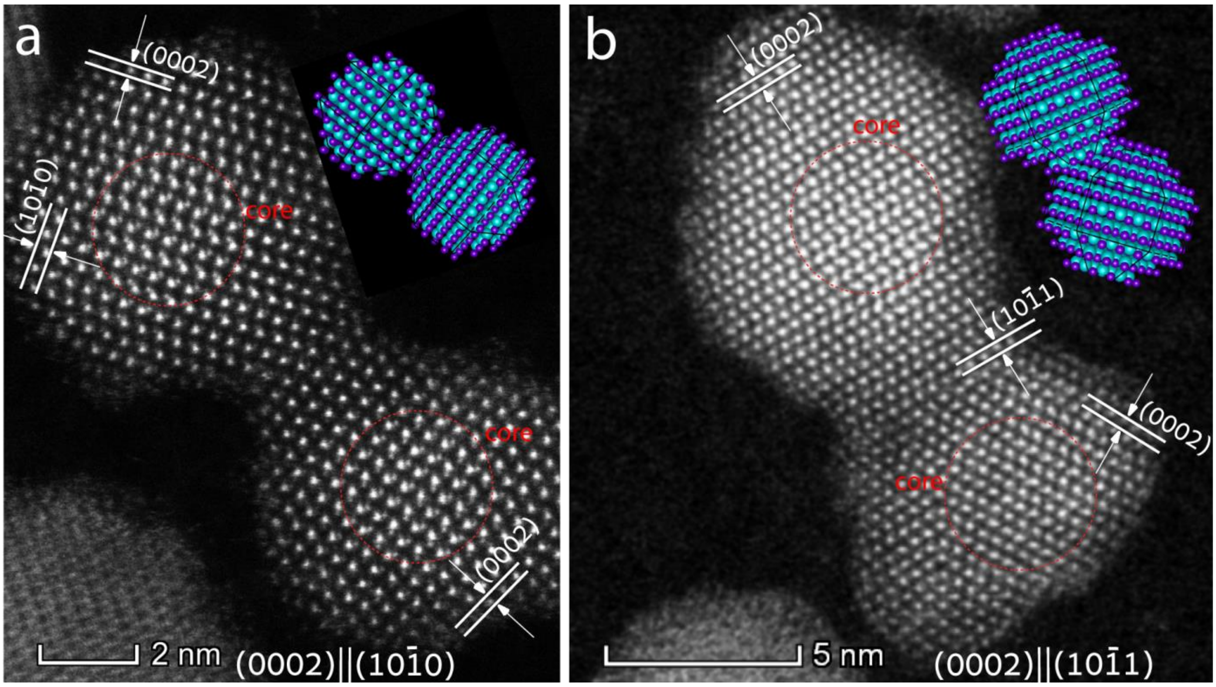

Supplementary Figure 16. HAADF-STEM images and atomic structure model of the fused 1.4/2.1 nm CdSe/CdS molecules with heteronymous-plane attachment on (0002)||($10\bar{1}0$) (a), (0002)||($10\bar{1}1$) (b). For the atomic model, the Cadmium atoms are marked in purple and Sulfur atoms in light blue.

Ensemble fluorescence lifetime measurements for 1.9/4.0 nm CQDs and the corresponding dimers

The molecule formed from larger CQDs (1.9/4.0 nm) also exhibit a quenching in the ensemble lifetime upon dimer formation but at a much lower extent compared with molecules of the small CQDs (1.4/2.1 nm). This is due to reduced tunneling and hybridization both because of the longer barrier and also due to the larger cores with less wavefunction spilling into the shell.

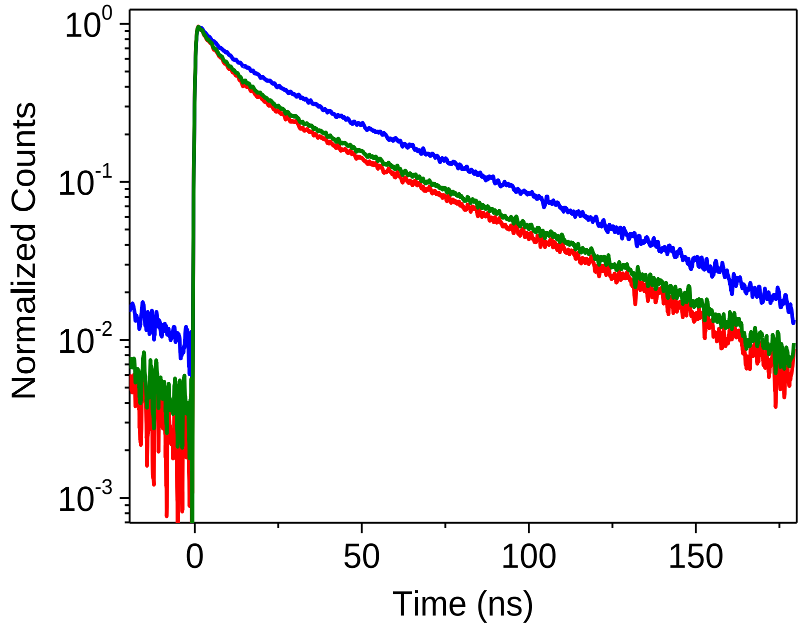

Supplementary Figure 17. Ensemble fluorescence lifetime decay curves of the monomer (blue), unfused dimer (green) and fused dimer (red) for 1.9/4.0 nm CQDs.

Single particle fluorescence lifetime for small 1.4/2.1 nm CQDs and the corresponding dimers

The fluorescence decay of a single 1.4/2.1 nm CQD follows mostly a mono-exponential decay of ~30ns or a bi-exponential decay with a small contribution from a 5 ns component. The average lifetime for the single dimer particles is clearly quenched and follows mostly a tri-exponential decay. The distribution of the average lifetime for dimer particles is shifted to short values, which is more pronounced for the fused dimers than for the unfused ones.

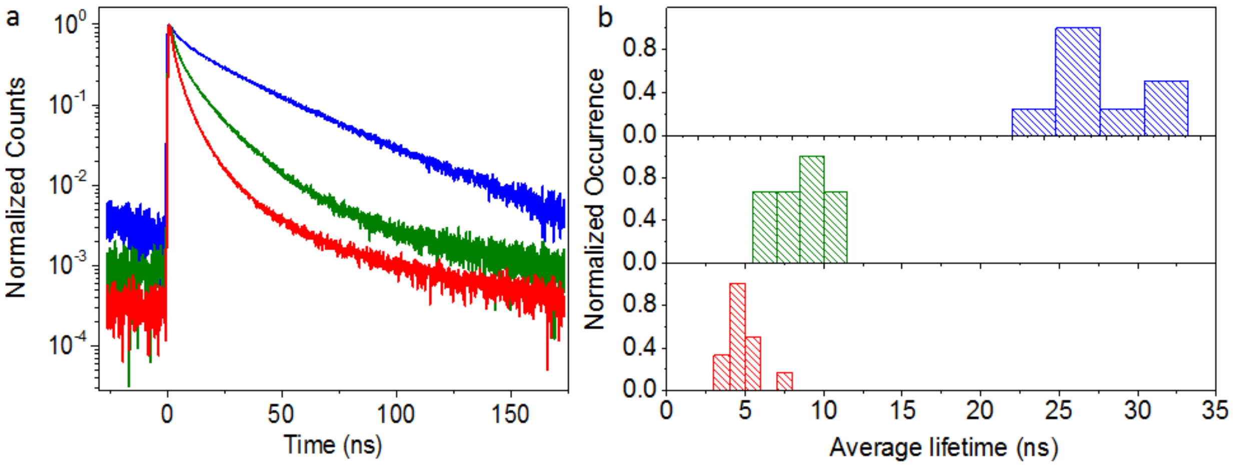

Supplementary Figure 18. Single particle fluorescence lifetime decay data for monomer (blue), unfused dimer (green) and fused dimer (red) composed of 1.4/2.1 nm CQD. (a) Representative single particle fluorescence lifetime decays. (b) Histograms summarizing the distribution of the average lifetimes for all three types of particles. Further shortening of the lifetime is observed upon fusion.

Characterization of the monomer particles that were treated with the fusion procedure:

The fraction of monomer particles found is in accordance with the size selective precipitation results and TEM analysis therein (~15%, Supplementary Figure 10). The optical properties of single monomer CQDs (Supplementary Figure 19-20) that although did not form a dimer, still were treated with the complete synthetic procedures of the fusion protocol, were characterized. We found that the monomer CQDs undergoing the fusion protocol retain their optical properties exactly as the unprocessed monomers, such as single exponential lifetime of the on state, on-off blinking of fluorescence and strong photon antibunching.

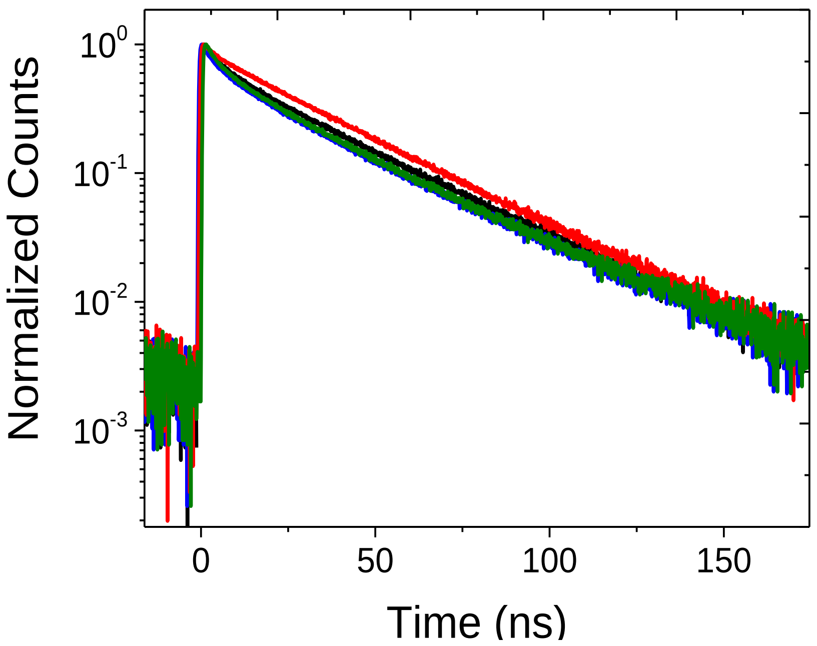

Supplementary Figure 19. Single particle fluorescence lifetime of a monomer CQD (1.4/2.1 nm) found along with the fused dimers. Two examples of the fusion protocol treated monomers are shown in blue and red curves. The black and green curves represent the lifetime of monomer CQDs that was not treated with the fusion procedure. The deviations of all representative average lifetimes are well under the distribution shown in Supplementary Figure 18.

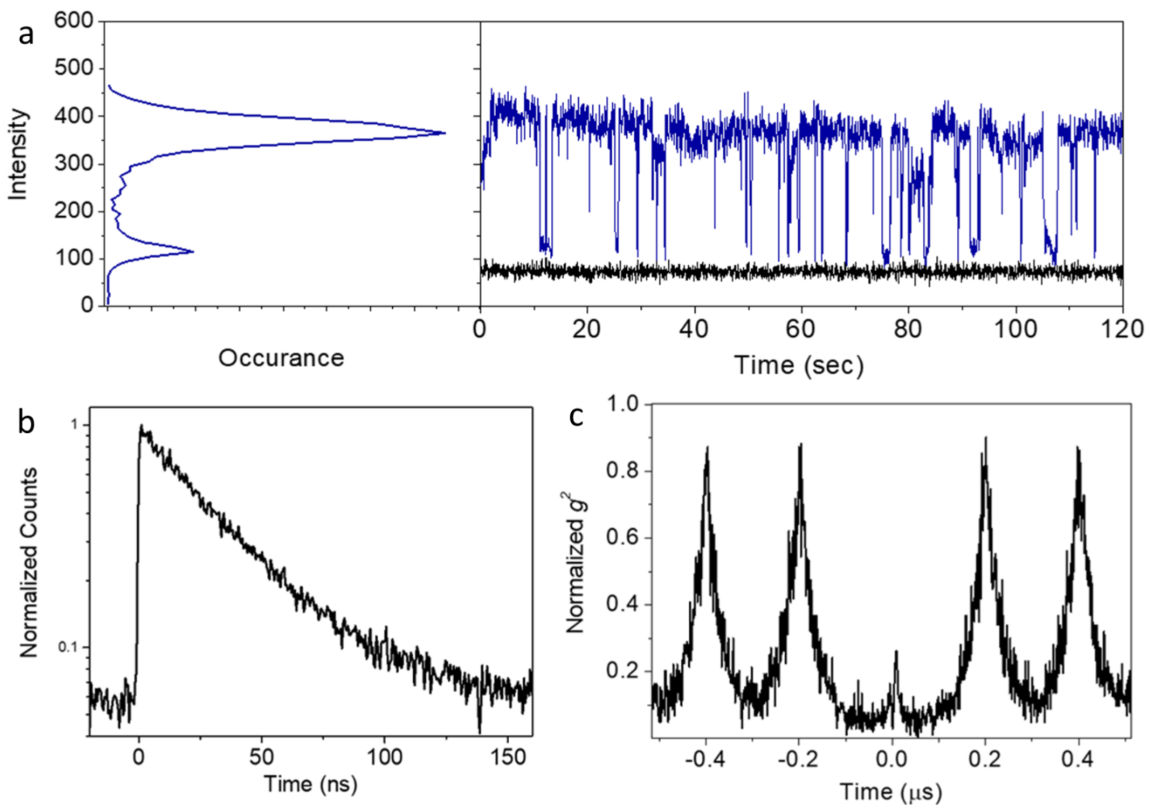

Supplementary Figure 20. Time-tagged time-resolved analysis of the fusion protocol treated monomer CQD (1.4/2.1 nm). (a) A bimodal on-off distribution of the intensity was found (bin-50 ms). The black curve represents the background noise. (b) Fluorescence lifetime of the on-state follows a single exponential decay of 31 ns. (c) Strong photon antibunching with g^2 value of 0.09. All these observations for the fusion protocol treated monomer are highly correlated with the untreated monomer particle as explained in the previous section.

Single particle fluorescence lifetime for 1.9/4.0 nm CQDs and the corresponding dimers

The shortening of the lifetime upon dimerization was also observed for the 1.9/4.0 nm CQDs. When compared with the lifetime distribution of the smaller particles (Supplementary Figure 18), the degree of shortening was found to be lower in this case. A similar type of distribution in the averaged lifetime for unfused and fused dimer indicates absence of significant additional coupling upon fusion, consistent with decreased tunneling-coupling in these molecules constructed from the larger CQDs.

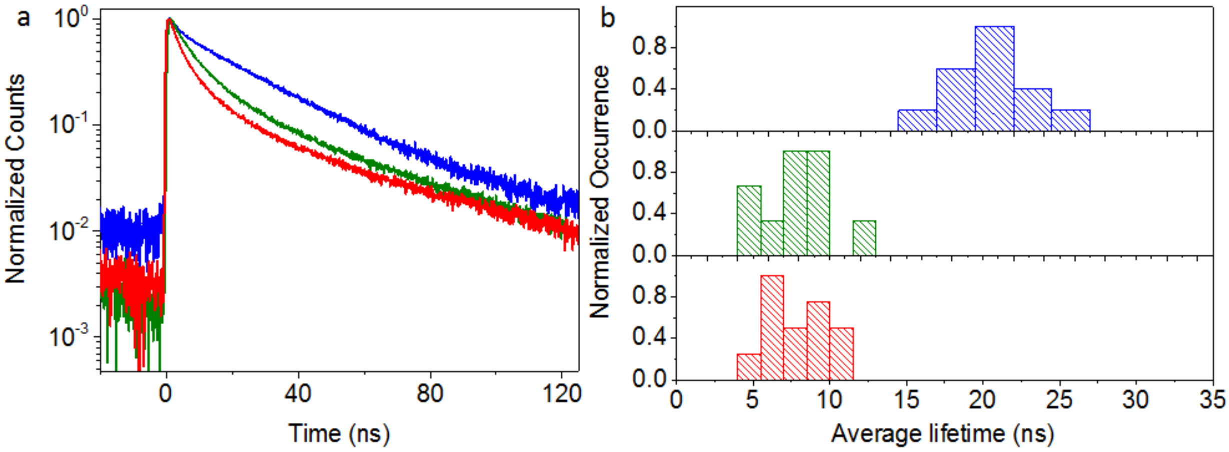

Supplementary Figure 21. Single particle fluorescence lifetime decay data for monomer (blue), unfused dimer (green) and fused dimer (red) composed of 1.9/4.0 nm CQD. (a) Representative single particle fluorescence lifetime decays (b) Histograms summarizing the distribution of average lifetimes for all three types of particles. For the shake of comparison, x-axis was kept the same as in Supplementary Figure 18.

Comparison of g^2 value for monomer and fused dimer

The antibunching at zero time delay accounts for the ratio of biexciton to exciton quantum yield (QY) $g^2 = \frac{Area_{(0ns)}}{Area_{(200\ ns)}} = \frac{QY_{(BX)}}{QY_{(X)}}$ when the value of <N> is kept well below 1. When the monomer (1.4/2.1 nm) and fused dimer were excited with similar <N> (~0.06) value, a much higher value of g^2 was obtained in the case of the fused dimer (0.68) than for the monomer (0.16) CQD. The possible pathways for the enhanced biexciton QY are explained in the main text.

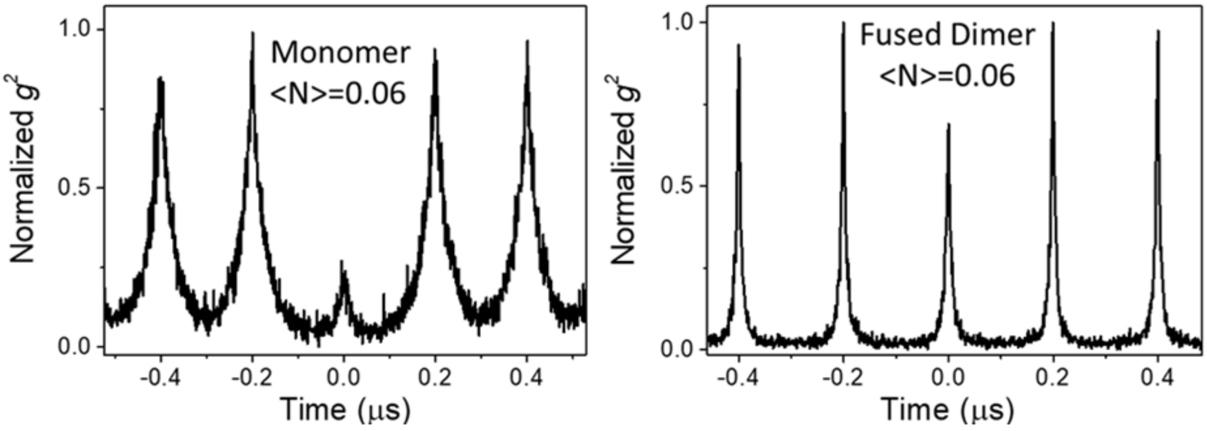

Supplementary Figure 22. Comparison of second order photon correlation at similar <N> value for excitation. The g^2 value were found to be 0.16 and 0.68 for the monomer and the fused dimer, respectively.

Attributes of fluorescence from single unfused dimer

The fluorescence from single unfused dimers also exhibited flickering nature instead of distinct on-off characteristics and followed a multi-exponential decay. The lifetime is not uniform throughout the intensity range, but rather presents distributions when analyzed at different intensity levels (Supplementary Figure 23(ii)). The single unfused dimer also gives rise to lower antibunching contrast.

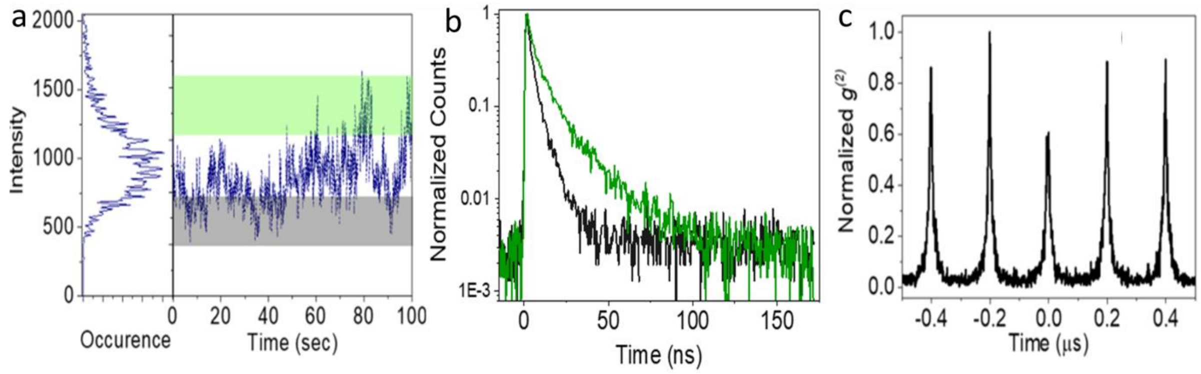

Supplementary Figure 23. Time-tagged time-resolved data for single unfused dimer comprised of 1.4/2.1 nm CQD(a) Photoluminescence time trace, (b) fluorescence intensity dependent lifetime (the lifetime curves bear the same color code as the shaded area from (a)), and (c) second order photon correlation (g^2).

Excitation intensity dependence of fluorescence flickering in dimer

The flickering nature of the fluorescence from a single fused dimer persists even in very low excitation conditions. The brown colored time trace in Supplementary Figure 24 was obtained in the lowest possible excitation (<N>~0.03), where the flickering of fluorescence occurs without reaching an off state (black curve). With increase in the excitation intensity an increment of the extent of flickering was observed along with shortening of the lifetime as described in Fig. 5f in the main manuscript.

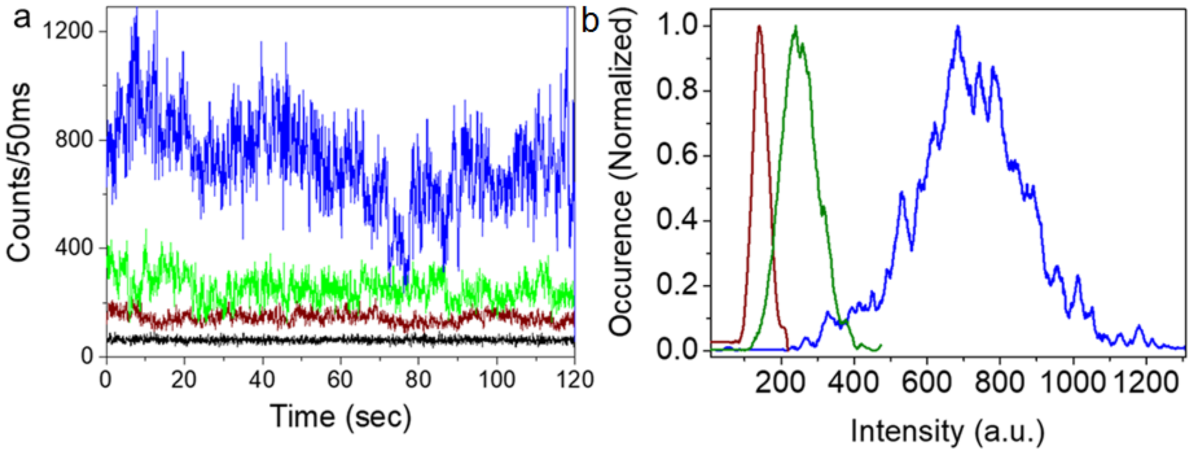

Supplementary Figure 24. Fluorescence time traces information with varying excitation intensity for a fused dimer comprised of 1.4/2.1 nm CQDs. The blue, green and brown curve represents the traces obtained from fused dimer with <N> value of 0.18, 0.09, 0.03, respectively. (a) Excitation fluence dependent PL time traces. The black curve represents the background noise in a similar configuration. (b) Intensity distribution of the traces in (a). Narrow distribution is observed with decreasing laser power.

Supplementary Table 2. The calculated and experimental monomer-to-dimer red shift for CdSe/CdS CQD molecules with different core/shell diameters.

**Core/Shell (nm)**	**Monomer emission peak (nm)**	**Fused emission peak (nm)**	**Red-shift (meV)-Exp**	**Red-shift (meV)-Calc**
**1.9/4.0**	637	637	0	0.65
**1.4/2.1**	603	606.5	11.8	11.3
**1.2/2.1**	593	597	13.9	12.8

Supplementary Table 3. The ensemble fluorescence lifetime values for different types of CQDs and corresponding homodimers (in toluene).

Core/Shell (nm)	Monomer			Unfused Dimer			Fused Dimer		
	$\tau_1(a_1)$	$\tau_2(a_2)$	τ_{avg}	$\tau_1(a_1)$	$\tau_2(a_2)$	τ_{avg}	$\tau_1(a_1)$	$\tau_2(a_2)$	τ_{avg}
**1.4/2.1**		25 (1.0)	25	7.3 (0.9)	30 (0.1)	8.7	5.2 (0.3)	9.7 (0.7)	9.7
**1.9/4.0**	8.9 (0.4)	39 (0.6)	24.5	7.9 (0.62)	38 (0.38)	17.5	8.1 (0.65)	39 (0.35)	17.1

τ_i values are represented in ns scale, a_i represents the weightage of a particular lifetime component

Supplementary References

1. S. Zhou, L. Dong, S. Popov, A. Friberg, Radiative properties of carriers in CdSe-CdS core-shell heterostructured nanocrystals of various geometries. *J. Eur. Opt. Soc.* **8**, 13042 (2013).

2. C. Grivas *et al.*, Single-mode tunable laser emission in the single-exciton regime from colloidal nanocrystals. *Nat. Commun.* **4**, 1–9 (2013).

3. O. Madelung, Semiconductors Data Handbook. Springer: Berlin, 2004.

4. M. P. Halsall, J. J. Davies, J. E. Nicholls, B. Cockayne, P. J. Wright, Photoluminescence of CdS/CdSe wurtzite superlattices and evidence for type II behaviour modified by piezoelectric fields. *J. Lumin.* **48**, 735-739 (1991).

5. G. Nair, J. Zhao, M. G. Bawendi, Biexciton Quantum Yield of Single Semiconductor Nanocrystals from Photon Statistics. *Nano Lett.* **11**, 1136–1140 (2011).

6. Z. Hens, I. Moreels, Light absorption by colloidal semiconductor quantum dots. *J. Mater. Chem.* **22**, 10406-10415 (2012).

7. C. Galland *et al.*, Two types of luminescence blinking revealed by spectroelectrochemistry of single quantum dots. *Nature*. **479**, 203–207 (2011).

8. C. Galland *et al.*, Lifetime blinking in nonblinking nanocrystal quantum dots. *Nat. Commun.* **3**, 908 (2012).

9. Y. Hu, Y. Sun, A Generic Approach for the Synthesis of Dimer Nanoclusters and Asymmetric Nanoassemblies. *J. Am. Chem. Soc.* **135**, 2213–2221 (2013).

10. K. Momma. & F. Izumi, VESTA 3 for three-dimensional visualization of crystal, volumetric and morphology data. *J. Appl. Crystallogr.* **44**, 1272-1276 (2011).

11. Z. D. Lu. *et al.* Direct Assembly of Hydrophobic Nanoparticles to Multifunctional Structures. *Nano Lett.* **11**, 3404-3412 (2011).
